# Cathepsin B inhibitor improves developmental competency and cryo-tolerance of in vitro ovine embryos

**DOI:** 10.1186/s12861-017-0152-2

**Published:** 2017-07-04

**Authors:** M. Pezhman, S. M. Hosseini, S. Ostadhosseini, Sh. Rouhollahi Varnosfaderani, F. Sefid, M. H. Nasr-Esfahani

**Affiliations:** 1 Department of Reproductive Biotechnology, Reproductive Biomedicine Research Center, Royan Institute for Biotechnology, ACECR, Royan St., Salman St., Khorasgan, Jey St, Isfahan, 8159358686 Iran; 2 Department of Biology, Faculty of Science, Nour Danesh Institute of Higher Education, Isfahan, Meymeh Iran

**Keywords:** Cathepsin B inhibitor (E-64), Ovine, Blastocyst, Apoptosis, Cryosurvival

## Abstract

**Background:**

Cathepsin B is a lysosomal cysteine protease involved in apoptosis and oocytes which have lower developmental competence show higher expression of Cathepsin B. Furthermore, expression of Cathepsin B show a decreasing trend from oocyte toward blastocyst stage.

**Results:**

Present study assessed the effect of cathepsin B inhibitor, E-64, on developmental competency and cryo-survival of pre-implantation ovine IVF derived embryos. Cathepsin B inhibitor was added during day 3 to 8 of development. One μM E-64 was defined as the optimal concentration required for improving blastocyst rate. This concentration also reduced DNA fragmentation and *BAX* as apoptotic markers while increasing total cell number per blastocyst and improving anti-apoptotic marker, the *BCL2*. We further showed that addition of 1.0 μM of E-64 during day 3 to 8 of development improved re-expansion and hatching rates of blastocysts post vitrification. E-64 also reduced rate of DNA fragmentation and *BAX* expression and increased total cell number per blastocyst and *BCL2* expression post vitrification. However, addition of E-64 post vitrification reduced the hatching rate.

**Conclusion:**

Therefore, it can be concluded that inhibition of cathepsin B in IVC, not only improves quality and quantity of blastocysts but also improves the cryo-survival of in vitro derived blastocysts.

## Background

Assisted reproductive technologies, such as in vitro fertilization (IVF) and intra-cytoplasmic sperm injection (ICSI), not only have transformed treatment of human infertility but also have had significant impact on farm animal reproduction and productivity. Among these techniques, embryos vitrification has progressed to become a useful adjunct technique, allowing storage of excess embryos for future use in human and animal embryo transfer programs. Despite intense research efforts and progress in field of vitrification, significant observable morphological and biochemical alterations are associated with vitrification. These alterations may lead to blastomere death and, eventually loss of embryo viability that are attributed to type and concentration of cryoprotectants [1], freezing protocol [2, 3], species, genotype, developmental stage and type of embryo [4, 5]. In this regard, some researchers have shown that the presence of dead cells is a common physiological finding in mammalian pre-implantation development and the number of dead cells is increased by vitrification [6].

Cell death in embryos has been mainly associated with programmed cell death or apoptosis [7] and is considered as potential cellular response to suboptimal developmental conditions and stress, such as vitrification [8, 9]. Numerous phenotypic phenomena including plasma-membrane blebbing, cell shrinkage, nuclear fragmentation, chromatin compaction, and chromosomal DNA fragmentation are associated with apoptosis [10]. Considerable researches have correlated these phenomena with low potential of embryos to reach blastocyst stage [11]. Therefore, improvement of culture media by different supplements to avoid apoptosis, is indispensable for production of good quality embryos in vitro.

Recent studies have shown that supplementation of culture medium with Rho-associated protein kinase inhibitor (ROCK inhibitor) [12], proteasome inhibitor [13], and antioxidants such as β-mercaptoethanol [14] can enhance and improve survival and quality of embryos produced in vitro.

Cathepsins are lysosomal cysteine proteases that play a crucial role in degradation of intracellular proteins in lysosomes [15]. They are also involved in induction of apoptosis through activating initiator caspases [16]. Indeed, it has been reported that sub-optimal pH and in vitro stressors can lead to secretion of cathepsins from lysosomes [17]. In this regard, cathepsin inhibitor (E-64) supplementation may overcome in vitro induced apoptosis and improve in vitro embryo development.

Inhibition of cathepsins improves in vitro developmental competency of embryos [18–25]. However, to our knowledge there is no report on the effect of cathepsin inhibitors on developmental competency of vitrified embryos. Therefore, aim of the current study was to assess the effect of culture supplementation with E-64, a cathepsin B inhibitor, on developmental competence, apoptosis and cryo-survival of in vitro produced ovine embryos.

## Methods

### Design of study

#### Experiment 1

To select the optimal concentration of E-64, on day 3 of culture during exchange of medium, embryos were randomly allocated to different concentrations (0.0, 0.1, 1.0, 10 μM) of E-64. Embryos were cultured in these concentrations of E-64 for up to 6 days. Percentage of morula, and blastocyst formed were determined on day 7–8 in four replicates.

#### Experiment 2

Following determination of optimal concentration of E-64, expanded blastocysts obtained from in vitro culture of embryos in the presence (IVC^+^) or absence (IVC^−^) of 1 μM of E-64 were vitrified and warmed. Vitrified/warmed blastocysts, here referred to as post warming (PW), derived from the after-mentioned groups (IVC^+^ or IVC^−^) were then randomly divided and cultured for 24 h in presence (IVC^−^/PW^+^, IVC^+^/ PW^+^) or absence (IVC^+^/ PW^−^, IVC^−^/PW^−^) of E-64. Cryo-survival (defined as rate of re-expansion) and hatching rates were determined and compared between groups. Finally, hatched blastocysts derived from each experimental group were used for mRNA analyses (3 replicates and minimum number of examined blastocysts in each replicate was 10), determination of cell number (3 replicates and minimum number of examined blastocysts in each replicate was eight) and assessment of DNA fragmentation by TUNEL assay (7 replicates and minimum number of examined blastocysts in each replicate was sixteen).

### Materials

Unless otherwise specified, all chemicals and media were obtained from Sigma Chemical Co. (St. Louis, MO, USA) and Gibco (Grand Island, NY, USA), respectively.

### In vitro embryo production

The procedure used for production of ovine embryos was according to Moulavi et al. [26]. Ovaries were obtained from a local abattoir and were transported to the laboratory in saline (15 °C–20 °C) and stored for additional 12 h at 15 C. COCs (cumulus-oocyte complexes) were isolated from 2 to 6 mm antral follicles with the aid of 20-G needles. Subsequently, they were washed with Hepes-supplemented tissue culture medium- 199 (HTCM199) + 10% FBS (fetal bovine serum). COCs with more than three layers of cumulus cells and homogenous cytoplasm were selected. Finally, 10 washed and selected COCs were cultured in 50 μl maturation medium [(MM: TCM199 + 10% FBS with 10 μg/ml FSH (follicle-stimulating hormone),10 μg/ml LH (luteinizing hormone), 1 μg/ml 17-beta estradiol, 0.1 mM cysteamine,10 ng/ml EGF (epidermal growth factor) and 100 ng/ml IGF1(insulin-like growth factor 1)] under mineral oil for 22–24 h at 38.5 °C, 5% CO2, in humidified air.

For in vitro fertilization (IVF), 100 μl of fresh sperm from a ram with proven fertility were kept under Tyrode’s albumin lactate pyruvate medium in 5% CO_2_, 38.5 °C, and humidified air for up to 45 min to allow motile sperm to swim up. After swim up, insemination was carried out by adding 5 × 10^3^ sperm/ matured COCs in fertilization medium containing NaCl 114 mM, KCl 3.15 mM, NaH_2_PO_4_, 0.39 mM, Na-lactate 13.3 mM, CaCl_2_ 2 mM, MgCl_2_ 0.5 mM, Na-pyruvate 0.2 mM, Penicillin 50 IU/ml, Streptomycin 50 μg/ml, NaHCO_3_ 25 mM, Heparin 10 μg/ml for 18–24 h at 38.5 °C under 5% CO_2_ in humidified air overlaid with light mineral oil.

On the next day, to remove the cumulus cells, the presumptive zygotes were vortexed in HTCM199 + FBS for 3 min. Then, they were cultured for 3 days in glucose and serum free modified synthetic oviductal fluid (mSOF) [27]. After the third day, cleaved embryos were transferred to mSOF in the presence of charcoal stripped serum (5%) and glucose (1.5 mM) for 5 days at 39 °C, 6% CO_2_, 5% O_2_ in humidified air under oil. Day 0 was defined as the day of fertilization. Therefore cleavage, blastocyst and hatching rates were determined on the day 3, 7 and 8 post embryo cultures.

### Vitrification and warming process

Vitrification–warming process was adopted from Martinez et al. [28]. Briefly, blastocysts were washed for 1 min in basic solution [BS: composed of phosphate buffer saline (PBS) and 20% FBS]. Then, blastocysts were equilibrated in equilibration solution (ES: 7.5% EG and7.5% DMSO in BS) for 5 min. Finally, they were exposed to vitrification solution (VS: 15% EG + 15% DMSO + 0.5 M sucrose) for 30 to 50 s and vitrified in minimal amount of vitrification solution on cryotops (Cryologic; CVM™, Fibreplug & Sleeve, Australia), which were quickly plunged into liquid nitrogen (LN_2_).

For warming, the cryotops were removed from LN_2_ and immediately tipped to warming solution 1 for 1 min (WS1: 1 M sucrose in BS) that was pre-equilibrated at 38.5 °C. Then, they were transferred for 3 min to warming solution 2 (WS2: 0.5 M sucrose in BS). Finally, blastocysts were allowed to remain in BS for 5 min and eventually co-cultured in mSOF in presence of charcoal stripped serum (5%) and glucose (1.5 mM) for 18-24 h. At this time, the percentages of re-expanded and hatched blastocysts were determined. Then blastocysts were used for evaluation of total cell number and DNA fragmentation.

### DNA-fragmentation

In Situ Cell Death Detection Kit (Promega Diagnostic Corporation, Mannheim, Germany), known as TUNEL (TdT-mediated dUTP-digoxigenin nick end labeling), was used for detection of apoptotic cells in blastocysts [10]. Hatched blastocysts were thoroughly washed in PBS + 1 mg/ml polyvinyl alcohol (PVA). Then, they were fixed in humid condition for 1 h at room temperature using 4.0% paraformaldehyde (*w*/*v*) in PBS. Following fixations, blastocysts were washed again in PBS/PVA and permeabilized for 30 min at room temperature, in 0.5% (*v*/v) Triton X-100 and sodium citrate. Next, permeable blastocysts were incubated for 10 min at room temperature in EQ buffer. Subsequently the blastocysts were incubated at 37 °C for 1 h in the dark under humid condition in TUNEL reaction mixture (equilibration buffer, nucleotide mix, and rTdT enzyme). Then, the blastocysts were allowed to remain in Buffer 2X for 15 min at room temperature. Eventually, the blastocysts were counterstained to label all nuclei with propidium iodide (PI) for 15 min, washed extensively in PBS, mounted on microscopic slides and observed under a fluorescence microscope (Olympus, Tokyo, Japan). Total numbers of nuclei were counted by PI. Cells were considered as TUNEL positive if their nuclei showed light green fluorescence against the background of PI (Fig. 2c).

### Differential staining

Differential staining was carried out according to Moulavi et al. [29]. Briefly, day 8 hatched blastocysts were washed in HTCM199 + 5 mg/ml BSA and permeabilized in 0.5% Triton X-100 in HTCM with 5 mg/mL BSA for 30 s. Then, blastocysts were transferred to 30 μg/ml of propidium iodide in basic medium and incubated for 10–20 s. Eventually, blastocysts were incubated for 15 min to 10 μg/ml Hoechst (H33342) at 4 °C, mounted in a drop of glycerol and observed under fluorescence microscope (Olympus, Tokyo, Japan). Inner cell mass (ICM) and trophectoderm (TE) numbers were distinguished based on their blue and red colors, respectively.

### Real time-PCR

Total RNA of blastocyst with the aid of the Micro-RNeasy kit (Qiagen, Canada) was extracted. For reverse transcription, 10 μl of total RNA was used in a final volume of a 20 μl reaction that contained 1 μl of random hexamer, 4 μl RT buffer (10×), 2 μl of dNTP, 1 μl of RNase inhibitor (20 IU), and 1 μl of reverse transcriptase (Fermentas, Canada). Reverse transcription was carried out at 25 °C for 10 min, 42 °C for 1 h and 70 °C for 10 min. Moreover, real Time-PCR was implemented using 1 μl of cDNA (50 ng), 5 μl of the SYBR Green qPCR Master Mix (2X) (Fermentas, Germany) and 1 μl of forward and reverse primers (5 pM) adjusted to a total volume of 10 μl using nuclease-free water. Real time PCR program was 1) 95 °C 4 min, 2) 94 °C 10 s, Ta 30 s, 72 °C 30 s, 40 cycles. To diminish the technical errors, Real Time-PCR was repeated three times. The transcripts abundance of *BCL2* and *BAX* were normalized to beta actin as reference gene (Table 1).

**Table 1:** Primer sequences

Gene symbol	Forward primer (5^′^-3^′^)	Reverse primer (5^′^-3^′^)	Annealing temp. (°C)
*BCL2*	CCTTCTTTGAGTTCGGAG	CCTTCAGAGACAGCCAG	61
*BAX*	AGCGAGTGTCTGAAGCG	CCCAGTTGAAGTTGCCGT	61
*β-actin*	CCATCGGCAATGAGCGGT	CGTGTTGGCGTAGAGGTC	59

### Statistical analysis

Data percentages were modeled to the binomial model of parameters by ArcSin transformation. Blastocysts rates, Real-time reverse transcription PCR data were examined using a one-way ANOVA followed by Tukey’s post hoc tests. For TUNEL staining, and differential staining, t-test was used. The differences were considered significant at *P* < 0.05. All data were presented as means ± S.E.M. and differences were considered as significant at *P* < 0.05.

## Results

### Effect of E-64 treatment on in vitro embryo production

**Fig. 1:**
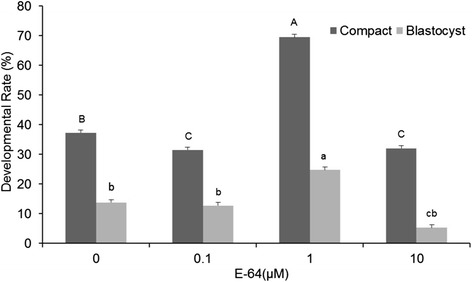
Effect of various concentrations of E-64 on developmental competence of ovine IVF embryos. Values in *columns* with *different letters* are significantly different (*P <* 0.05). *Uppercases* and *lowercases* denote significance for compact morula and blastocyst rate, respectively

As shown in Fig. 1, in the first experiment to investigate appropriate concentration of E-64, different concentrations (0, 0.1, 1.0, and 10 μM) of E-64 were added to IVC medium on day 3 in which embryos were cultured for 6 days. Addition of 1.0 μM E-64 from day 3 to 8 significantly increased compaction rates compared to control (69.5 ± 2.9% vs. 37.25 ± 2.13%, respectively; *P* < 0.05). The percentage of embryos that developed to the blastocyst stage in 1.0 μM (24.75 ± 3.1%) was also significantly higher than that of embryos cultured with 0.1, 10 μM and control group (12.75 ± 2.4, 5.25 ± 0.9, 13.75 ± 1.1, respectively; *P* < 0.05).

Hatching rates were 7.5 ± 1.29, 4.75 ± 2.06, 11.5 ± 2.88%, 1.25 ± 0.75 in 0.0, 0.1, 1.0 and 10.0 μM E-64, respectively. Hatching rate was significantly higher in 1.0 μM compared to other groups (*P* < 0.05).

**Fig. 2:**
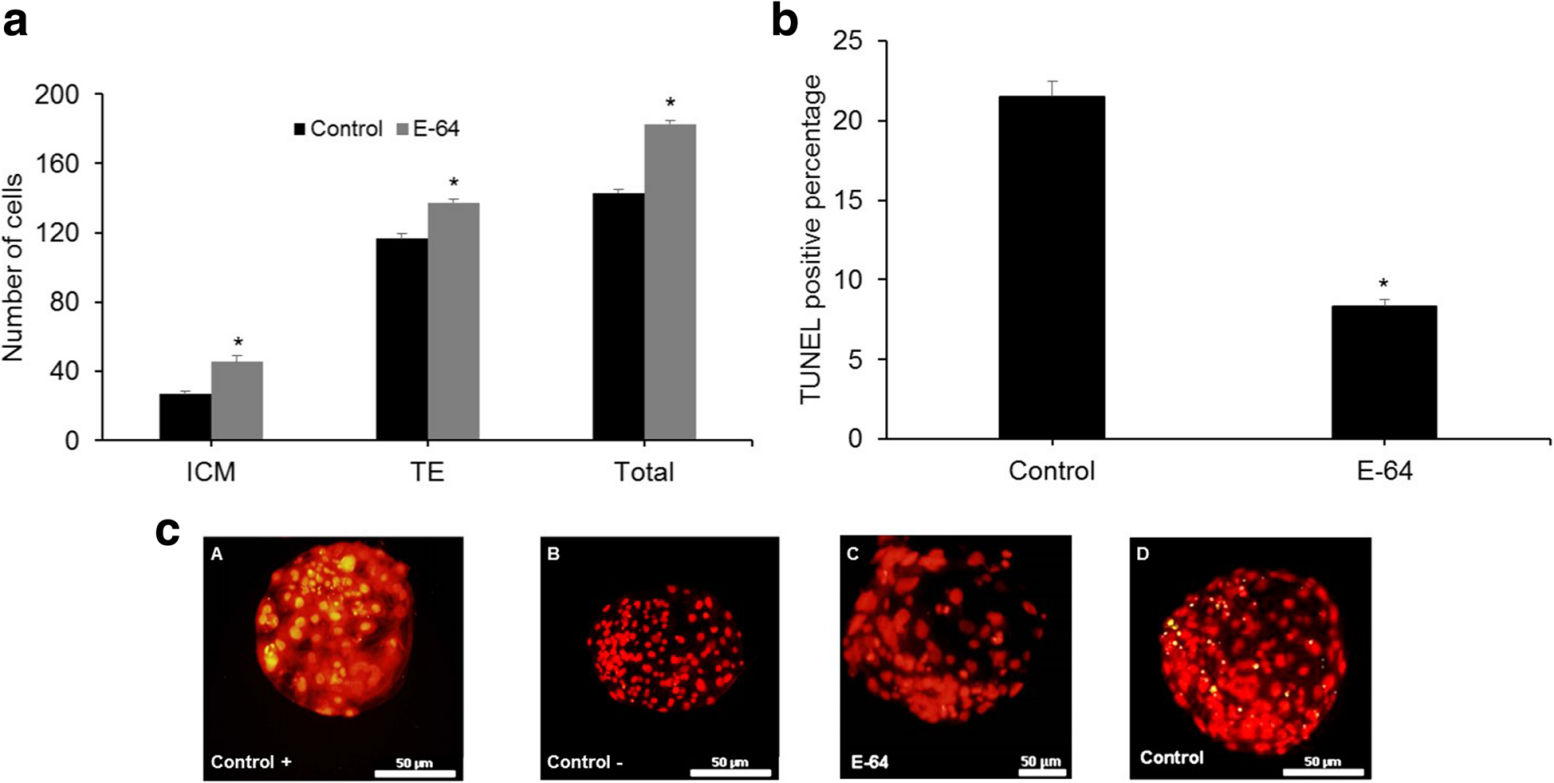
Effect of E-64 (1 μM) during embryo culture medium on **a** blastocyst total cell number and **b** percentage of DNA fragmentation assessed by TUNEL assay. Mean values with asterisk denote significant difference at *P <* 0.05 compared to control. **c** TUNEL-positive cells that appeared in yellow-green. Scale bar represents 50 μm

Moreover, for analysis of quality of hatched blastocysts, differential staining of the blastocysts showed that E-64 supplementation significantly affected total cell number of blastocyst (183 ± 1.6 vs. 143.16 ± 1.6; *P* < 0.05; Fig. 2a). Also inner cell mass (ICM) and trophectoderm (TE) cell number in presence of 1.0 μM E-64 were significantly higher than the control group (ICM: 26.75 ± 1.84 vs. 45.4 ± 3.93; TE: 116.42 ± 3.11 vs. 137.52 ± 1.67; *P* < 0.05).

Furthermore, the rate of TUNEL-positive cells of blastocyst derived from the E-64-treated group were significantly lower than the control group (8.34 ± 0.4% vs. 21.4 ± 0.9%, *P* < 0.05, Fig. 2b).

### Effect of E-64 on post-warming survival of vitrified blastocysts

**Fig. 3:**
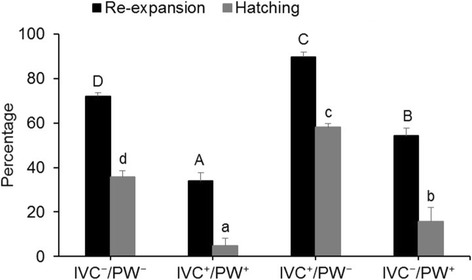
Effect of E-64 supplementation during in vitro culture and/or post warming on re-expansion and hatching rates of blastocyst. *Columns* with *different letters* are considered as significant (*P <* 0.05). *Uppercases* and *lowercases* denote significance for re-expansion and hatching rate, respectively. IVC^+^ means embryos cultured in the presence of 1 μM E-64, while IVC^−^ means embryo cultured in absence of E-64. PW^+^ and PW^−^ refers to presence or absence of 1 μM E-64 after warming, respectively

In vitro re-expansion and hatching rates of blastocyst after vitrification/warming are shown in Fig. 3. In this regard, the proportion of survived and also hatched vitrified/warmed blastocysts in the experimental groups (IVC^+^/PW^+^, IVC^−^/PW^+^, IVC^+^/PW^−^, IVC^−^/PW^−^) were (33.9 ± 3.8% and 4.9 ± 3.2%), (54.2% ± 3.4 and 15.8 ± 6.1%), (89.7% ± 2.1% and 58.1 ± 1.7%), and (72.1 ± 1.4% and 35.8 ± 2.6%) which were significantly (*P* < 0.05) different between groups (Fig. 3).

**Fig. 4:**
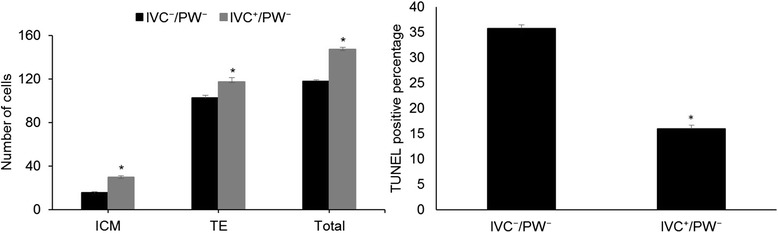
Comparison of **a** total cell number and **b** percentage of nuclei with DNA fragmentation in blastocysts derived from IVC^+^/PW^−^ and IVC^−^/PW^−^ groups. Mean values with *asterisk* denote significant difference at *P <* 0.05

Analysis of TCN of cryopreserved blastocysts indicated significant differences between the two groups [IVC^+^/PW^−^ (147 ± 2) compared to IVC^−^/PW^−^ (118 ± 1), Fig. 4a]. Also ICM and TE cell number in the IVC^+^/PW^−^ group was significantly higher than the control group (ICM: 15.45 ± 1.0 vs. 29.76 ± 1.08; TE: 102.41 ± 2.65 vs. 117.28 ± 3.56; *P* < 0.05).

Furthermore, TUNEL assessment of the cryopreserved blastocysts clearly revealed a significant difference in the percentage of TUNEL-positive cells between two groups [IVC^+^/PW^−^ (15.9 ± 0.6%) compared to IVC^−^/PW^−^ (35.7 ± 0.6%), Fig. 4b; *P* < 0.05].

Within E-64 supplemented groups, IVC^+^/PW^−^ induced the best cryo-protection.

### Effect of E-64 on expression of apoptosis-related genes before and after vitrification

**Fig. 5:**
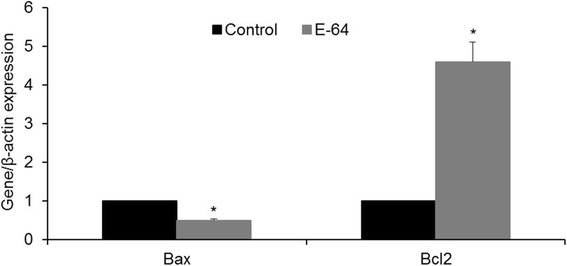
Effect of E-64 (1 μM) during in vitro culture on relative expression of *BAX* and *BCL2*, in ovine IVF blastocysts. *Asterisks* indicate statistically significant differences from control (*P <* 0.05)

**Fig. 6:**
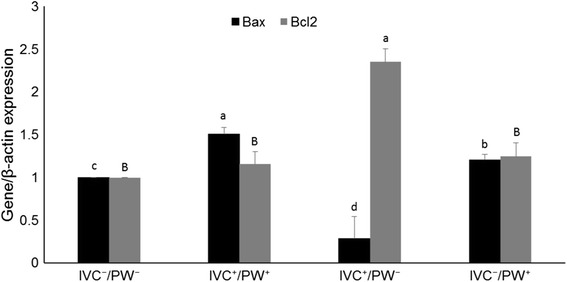
Effect of E-64 on in vitro culture and/or post warming on relative expression of *BAX* and *BCL2*, in ovine IVF blastocysts. Values in *columns* with *different letters* are significantly different (*P <* 0.05)

Figure 5 shows that the expression of anti-apoptosis-related gene, *BCL2* was significantly higher in blastocysts from E-64 treatment than the control (*P* < 0.05) while the expression of pro-apoptotic gene, *BAX* was significantly less in blastocysts from E-64 treatment groups than in the control (*P* < 0.05). Interestingly, after vitrification/warming, expression of *BCL2* significantly increased in IVC^+^/PWˉ compared to IVC^−^/PW^−^ (*P* < 0.05). Conversely, expression of *BAX* in IVC^+^/PW^−^ was significantly lower than IVC^−^/PW^−^ (*P* < 0.05, Fig. 6).

## Discussion

Lysosomes are specialized intracellular organelles and play indispensable role in many physiological functions including: endocytosis, phagocytosis, and autophagy [30–32]. In addition, in presence of exogenous stresses such as altered pH and heat shock or suboptimal culture conditions [17], lysosomal cysteine proteases, like cathepsin B, are released from lysosomes. Cathepsins can induce apoptosis directly through initiator caspases or indirectly via release of cytochromes from mitochondria and eventually leads to completion of apoptosis via activation of effector caspases [33].

Increased expression of cathepsin B is inversely correlated with quality of cumulus-oocyte complexes (COCs) in cattle [7]. Furthermore, expression of cathepsin B is higher in poor quality bovine oocytes as compared to good-quality ones [22]. In this regard, Balboula et al. showed that addition of 1 μM E-64 during in vitro maturation (IVM), improves quality of COCs and their developmental competency. These authors also showed that heat shock stress increases the expression of cathepsin B and its inhibition by E-64 reduces injuries due to heat stress [34].

It has also been reported that E-64 treatment after IVF followed by IVC for 6 days significantly improved developmental competences and increased number of good quality bovine embryos [23]. On the other hand, Min et al. (2014) reported that treatment during IVC with E-64 (0.1 and 0.5 μM) significantly improves developmental rates without any noticeable effect on cleavage rate [24]. Unlike the former study which assessed role of cathepsin B inhibition during IVM, other research groups showed that addition of E-64 during in vitro culture (IVC) also improves rate and quality of derived blastocysts [7, 22, 24, 34]. Both these studies concluded that addition of E-64 to IVC medium has no effect on early cleavage rates. Based on these result, we assessed the effect of E-64 post maternal embryonic transition on quantity and quality of ovine derived blastocysts. In agreement with previous studies, this study also revealed that among assessed concentrations, 1.0 μM E-64 significantly improves rates of ovine embryo compaction and blastocyst formation. In addition, we showed that rate of apoptosis, assessed by TUNEL, was significantly reduced by treatment with E-64. Since cathepsin B mediates its indirect effect through mitochondria intrinsic pathway, we also assessed the relative expression of pro-apoptotic (*BAX*) and anti-apoptosis (*BCL2*) related genes which play an important role in regulating cell death by controlling release of cytochrome-c into cytosol [35]. As depicted in Fig. 5, addition of E-64 increased expression of *BCL2* and reduced expression of *BAX*, indicating that E-64 can limit apoptosis induced by sub-optimal culture conditions.

The second point highlighted in this study was the link between developmental competence and vitrification in ovine embryos. During vitrification, embryo exposure to a highly-concentrated solution of cryo-protectants leads to stress or injuries to membranes, cellular organelles and release of cathepsin B from lysosomes [36–41]. Moreover, the sensitivity of embryos to cryopreservation is closely related to culture conditions [8, 9]. Therefore, in this study, we evaluated the effect of addition of E-64 during day3 to day8 on cryosurvival of derived blastocysts.

In results depicted in Fig. 3, addition of E-64 to culture medium during embryonic development enhances the overall re-expansion and cryo-viability of the blastocysts. However, the difference for rate of blastocyst re-expansion became significant when E-64 was added to IVC before vitrification during day 3 to 7 (90% ± 2% IVC^+^/PW^−^) compared to control (IVC^−^/PW^−^) or when E-64 was added before and after vitrification (IVC^+^/PW^+^). These data are consistent with the interpretation of positive effect of E-64 addition to IVC. It is very likely that addition of E-64 leads to production of more competent embryos with better cryosurvival potential, which was further confirmed by assessment of percentage of apoptotic cells, total cell number and expression of pro- and anti-apoptotic genes. In contrast, the data indicate that addition of E-64 post warming has a negative effect on the rate of re-expansion. The rate of re-expansion is significantly lower when E-64 was used after warming (IVC^−^/PW^+^ or IVC^+^/PW^+^) compared to its absence before and after vitrification (IVC^−^/PW^−^). This observation raises the questions; could cathepsin B have a role in blastocyst re-expansion or is this effect due to toxic effect of high concentration of E-64? Indeed, it is know that permeability of embryos is highly altered through cryopreservation. Therefore, could the optimal concentration be toxic post vitrification, as higher concentration of E-64 (10 μM) reduced the developmental competency. Therefore, further experiment and optimization is needed to define the concentration of E-64 required after vitrification.

The overall improved effect observed by E-64 treatment can be explained by direct and indirect mechanism of action of cathepsin B. It is likely, exposure to cryo-protectant or reactive oxygen species (ROS) produced during cryopreservation, may directly activate Type II class, initiator caspases. Alternatively, cryopreservation may lead to release of cathepsin B from lysosomes and induce mitochondrial membrane degradation, a condition known as permeability transition. This effect leads to the release of pro-apoptotic factors into the cytosol. In this regard, Balboula et al. has shown that heat stress in oocytes leads to a defect in lysosomal membrane permeability which results in lysosomal aggregation and release of cathepsin B into the cytosol [34]. Kim et al. evaluated localization of cathepsin B and cytochrome C in presence of E-64 and showed co-localization of these factors in porcine embryos [25]. In both bovine and porcine embryos, these observations were reversed by treatment with E-64. E-64 decreases both the activity of caspase 3 and its mRNA while decreasing only the activity of cathepsin B. It is also important to note that cathepsin B, on its own, can also induce nuclear apoptosis independent of caspase 3 [42, 43] and ablation of cathepsin B makes cells more resistant to inflammation induced apoptosis [44].

Similarly, rates of hatching were significantly reduced by adding E-64 post warming, while its addition during IVC significantly improved the hatching rate compared to control (IVC^−^/PW^−^). It is noteworthy that blastocysts hatch by their intrinsic ability to produce zonalytic factor(s) that have cysteine protease-like activity. In this regard, several proteases are expressed before hatching [45, 46] and they play important role in this process [47–53]. Therefore, it is likely that E-64 inhibits these proteases involved in hatching and this may explain the reduced rate of hatching by presence of E-64 post warming. Indeed, the rate of hatching was also reduced in all concentration of E-64 during IVC compared to control, except at 1 μM concentration. The improved higher hatching rate at 1 μM E-64 is very likely related to intrinsic effect of E-64 to improve the quality of derived blastocysts. The ability of E-64 to reduce hatching rate also indicate that cathepsins are likely to be involved in ovine blastocyst hatching.

## Conclusion

In conclusion, results of this study indicated that supplementation of IVC media with 1 μM E-64, an exogenous inhibitors of Cathepsins, improves quality and quantity of blastocyst formation. Furthermore, addition of E-64 during IVC also improve rate of re-expansion and hatching post vitrification. However, addition of 1 μM E-64 to media post vitrification/warming has a negative effect on embryo re-expansion and hatching rates. This effect may be related to toxic concentration of E-64 which is likely to be related to altered membrane permeability post vitrification. Moreover, negative effect of E-64 after warming can be related to incomplete blastocyst hatching because of interference in secretion of zonalytic proteases which requires further investigation.

## Abbreviations

6-DMAP: 6-dimethyl aminopurine
BS: Basic medium
CPA: Cryoprotectant
DMSO: Dimethyl sulfoxide
EG: Ethylene glycol
ES: Equilibration solution
IVF: In vitro fertilization
LN2: Liquid nitrogen
MM: Maturation medium
PBS: Phosphate-buffered saline
ROCK: Rho-associated protein kinase
TCM-199: Tissue culture medium-199
VS: Vitrification solution
